# Genomic analyses identify nosocomial transmission of ST23 carbapenem-resistant hypervirulent *Klebsiella pneumoniae* mediated by a conjugative IncFII_K2_ NDM-1 plasmid

**DOI:** 10.1080/21505594.2026.2668167

**Published:** 2026-05-03

**Authors:** Tao Chen, Xueting Wang, Luying Xiong, Yan Geng, Hui Ding, Yunbo Chen, Ping Shen, Yonghong Xiao

**Affiliations:** a State Key Laboratory for Diagnosis and Treatment of Infectious Diseases, National Clinical Research Center for Infectious Diseases, China-Singapore Belt and Road Joint Laboratory on Infection Research and Drug Development, National Medical Center for Infectious Diseases, Collaborative Innovation Center for Diagnosis and Treatment of Infectious Diseases, The First Affiliated Hospital, Zhejiang University School of Medicine, Hangzhou, Zhejiang, China; b Department of Clinical Laboratory, The Second Affiliated Hospital of Xi’an Jiaotong University, Xi’an, Shaanxi, China; c Department of Clinical Laboratory, Lishui Municipal Central Hospital, Lishui, Zhejiang, China

**Keywords:** Klebsiella, ST23, hypervirulence, plasmid-mediated resistance, NDM-1, IncFII_K2_

## Abstract

The World Health Organization has identified ST23 carbapenem-resistant hypervirulent *Klebsiella pneumoniae* (CR-hvKP) as a critical public health threat. Through China’s national surveillance system (BRICS), we identified and characterized ST23 CR-hvKP bloodstream isolates from 2019–2023. Among 1069 CRKP bloodstream isolates, four ST23-K1 strains (0.37%) were detected across two hospitals, including a nosocomial transmission pair (differing by only 2 core SNPs). Clinical outcomes revealed that three of the four patients achieved recovery following appropriate antibiotic therapy, with one mortality case attributed to underlying comorbidities. All four ST23 isolates demonstrated resistance to multiple antibiotics, indicating a pattern of multidrug resistance. Genomic analysis uncovered diverse resistance mechanisms: the nosocomial transmission pair possessed conjugative IncFII_K2_ NDM-1 plasmids, while the others harbored conjugative IncFII_K34_ KPC-2 plasmids, which exhibited reduced carbapenem resistance attributed to the downregulation of *bla*_KPC-2_ expression. Conjugation assays revealed high transferability (10^−5^ for IncFII_K2_ NDM-1; 10^−4^ for IncFII_K34_ KPC-2). Whole-plasmid comparative genomics analysis suggested that the IncFII_K2_ NDM-1 plasmids shared > 99% identity with historical IncFII_K2_ plasmid backbones from Chinese *K. pneumoniae* isolates (2014–2022), suggesting local evolution. Notably, only 4.4% (26/590) of global IncFII_K2_ plasmids carried *bla*_NDM-1_ and all IncFII_K2_ NDM-1 plasmids maintain conjugative potential. All strains harbored conserved virulence plasmids and maintained hypervirulence, as indicated by a mouse infection model, with the exception of one strain that exhibited *cps* mutations. This study reports the first genomic evidence of a high-frequency conjugative IncFII_K2_ NDM-1 plasmid in a hospital-transmitted ST23 CR-hvKP clone, highlighting the need for plasmid-focused surveillance to control this potential threat.

## Introduction

Hypervirulent *Klebsiella pneumoniae* (hvKP) is capable of infecting both immunocompetent and immunocompromised individuals, with a high propensity for invasive infections [1]. A growing concern is the rise of carbapenem-resistant hvKP (CR-hvKP) with resistance to nearly all clinically used β-lactams, severely restricting therapeutic options [2]. On 31 July 2024, the World Health Organization had issued a report on the global status of hvKP infections, noting a significant increase in the detection of ST23 CR-hvKP isolates [3]. However, due to inadequate laboratory capacity for strain identification, the true global burden of both hvKP and CR-hvKP is likely substantially underestimated.

ST23 CR-hvKP typically arises through the acquisition of carbapenemase-encoding plasmids by ST23 hvKP strains [4]. Although several factors are thought to restrict the formation and dissemination of CR-hvKP – such as the hypermucoviscosity and thick capsule of hvKP, which may impede plasmid uptake – multiple carbapenemase-encoding plasmids have been identified in recent global ST23 CR-hvKP clones [5–7]. For instance, an IncL-type plasmid carrying OXA-48 was detected in an ST23 CR-hvKP strain from Taiwan in 2018 [8], while an IncFII_K2_ KPC-2 plasmid was identified in a U.S. isolate in 2019 [9], and an IncP1 KPC-2 plasmid was reported in mainland China in 2021 [10]. Additionally, Jiang et al. identified the IncFII_K34_ plasmid harboring *bla*_KPC-2_ as a key driver of the global emergence of ST23 CR-hvKP [11]. Nevertheless, these reports remain sporadic, and further surveillance is needed to assess the role of novel plasmid types and carbapenemases in the spread of ST23 CR-hvKP.

To our knowledge, reports on nosocomial transmission of ST23 CR-hvKP causing bloodstream infections are limited. Here, we demonstrate the emergence of hospital-transmitted ST23 CR-hvKP bloodstream infection isolates carrying a conjugative NDM-1-producing IncFII_K2_ plasmid. Although IncFII_K2_ KPC-2 plasmids are established contributors to the dissemination of carbapenem-resistant *Klebsiella pneumoniae* (CRKP) clones ST11 and ST258 [12], our findings demonstrate that IncFII_K2_ NDM-1 plasmids, while exhibiting high conjugation frequency, have been scarcely documented and have not been previously associated with the development of ST23 CR-hvKP strains. This finding suggests an alternative resistance transmission pathway that warrants further investigation.

## Materials and methods

### CRKP isolates and whole genome sequencing

We performed a retrospective analysis of 1069 CRKP isolates collected from bloodstream infections between 1 January 2019 and 31 December 2023. These isolates were obtained from 72 hospitals across 22 provinces as part of the national surveillance framework (Blood Bacterial Resistance Investigation Collaborative System, BRICS) in China. All participating hospitals sent their isolates to the central laboratory quarterly. Carbapenem resistance was defined as minimum inhibitory concentration (MIC) values ≥ 4 μg/mL for imipenem or meropenem, or ≥2 μg/mL for ertapenem [13]. All CRKP isolates were identified using matrix-assisted laser desorption ionization time-of-flight mass spectrometry (MALDI – TOF MS) at First Affiliated Hospital of Zhejiang University. The sample collection protocol was approved by the institutional review board of the First Affiliated Hospital of Zhejiang University in China.

Genomic DNA from CRKP isolates was extracted using SteadyPure Bacterial Genomic DNA Extraction Kit (Accurate Biology, Changsha, China). The genomes were sequenced using the Illumina HiSeq X-Ten platform with a 2 × 150 bp paired-end read.

### Genome assembly and sequence typing

Whole-genome assembly was performed using SPAdes (version 3.10.0) [14] with Illumina short-read sequencing clean data. In silico multilocus sequence typing (MLST) was performed using mlst (https://github.com/tseemann/mlst). The capsular polysaccharide (KL) and lipooligosaccharide (OL) loci were identified via Kleborate (version 2.0.147) [15].

### Clinical information collection

We retrieved and reviewed the medical records from local sites. A standardized surveillance form captured comprehensive epidemiologic and clinical data, including: demographic characteristics (age, sex), admission diagnosis, comorbidities, invasive procedures performed, infection acquisition sources, laboratory findings, antimicrobial regimens, hospitalization duration, and clinical outcomes. A hospital-acquired infection was defined as any microbiologically confirmed infection that manifested ≥ 48 hours after hospital admission, with no evidence of incubation at the time of admission. This study was approved by the Research Ethics Committee of the First Affiliated Hospital, Zhejiang University School of Medicine (2024–488).

### Genome annotation and SNP analysis

Whole-genome sequencing was further conducted using the Nanopore MinION platform (Oxford Nanopore Technologies, Oxford, UK). For each ST23 isolate, a hybrid assembly approach combining both Illumina short reads and Nanopore long reads was performed using Unicycler (version 0.4.0) [16] to generate complete chromosomal and plasmid sequences. We detected the coding sequences (CDSs) from complete genome assemblies via Prokka (version 1.13.0) (https://github.com/tseemann/prokka). AMRFinderPlus (version 4.0.3) was employed to identify resistance genes in the genome [17]. Plasmid replicon genes were initially detected using PlasmidFinder 2.1 (https://cge.food.dtu.dk/services/PlasmidFinder) with default parameters and were subsequently typed at the allele level via pMLST (https://pubmlst.org/organisms/plasmid-mlst). In silico predictions of the plasmid transferability was performed using MOB-suite (version 3.1.9) (https://github.com/phac-nml/mob-suite). BLASTN was performed to screen for sequences homologous to the sequenced plasmids in the NCBI database. Comparison between homologous plasmids was conducted using EasyFig (version 2.2.2) [18]. To characterize the virulence profiles of the isolates, we detected virulence-associated genes using ABRicate (version 1.0.0) (https://github.com/tseemann/abricate) with the VFDB database [19]. BRIG (version 0.95) was used to compare and visualize structural variation between virulence plasmid sequences [20].

Each strain sequence was mapped to NTUH-K2044 (GCF_000009885.1) using Snippy (version 4.6.0) (https://github.com/tseemann/snippy). Gubbins (version 2.4.1) [21] was used to eliminate recombination and obtain core single-nucleotide polymorphisms (cSNPs) and multiple sequence alignments.

### Antimicrobial susceptibility testing

Antimicrobial susceptibility testing was conducted on all isolates following Clinical and Laboratory Standards Institute (CLSI) guidelines [13], with MICs of common antibiotics determined by the agar dilution method. For tigecycline and polymyxin B, MICs were determined by broth microdilution following European Committee on Antimicrobial Susceptibility Testing (EUCAST) recommendations, with interpretation according to EUCAST guidelines (https://www.eucast.org/).

### RTq-PCR

Total RNA was extracted from *bla*_KPC-2_-positive *Klebsiella pneumoniae* strains ATCC BAA-1705 (control), KP245861, and KP245905 during mid-to-late logarithmic growth phase using the QIAGEN RNeasy Mini Kit. RNA concentration and purity were assessed using a NanoDrop ND-2000 spectrophotometer (Thermo Scientific). cDNA synthesis was performed with the PrimeScript™ RT Reagent Kit (Takara Bio, China). Relative expression of the *bla*_KPC-2_ gene was verified by RT-qPCR on a CFX96 Real-Time PCR System (Bio-Rad), with normalization to the *gapA* reference gene using the 2^−ΔΔCt^ method [22]. The following primers were used: gapA-F (5’- GAAAGGCGTTCTGGGTTAC), gapA-R (5’- GATGTGGGCAATCAGATCC), KPC-F (5’-GCGGCAGCAGTTTGTTGATT), and KPC-R (5’-CGGCATAGTCATTTGCCGTG).

### Conjugation and fitness evaluation

Conjugation experiments were performed using rifampicin-resistant *Escherichia coli* EC600 as the recipient strain. Donor and recipient cells (each at 10^8^ CFU/mL in logarithmic phase) were mixed at a 1:1 ratio (500 μL total volume) and co-incubated overnight at 37°C on LB agar plates with or without meropenem (0.01 μg/mL). Transconjugants were selected on LB agar supplemented with meropenem (0.2 μg/mL) and rifampicin (100 μg/mL). Putative transconjugants were confirmed by PCR amplification of carbapenemase genes (or plasmid replication genes) as genetic markers. Conjugation frequency was calculated as the number of transconjugants per recipient cell. However, because KP169509 harbored two distinct conjugative NDM-1-encoding plasmids, conjugation frequency could not be accurately determined for this isolate. The fitness cost was assessed by conducting a growth curve assay on carbapenemase plasmid-carrying transconjugants. Briefly, bacterial suspensions (10^8^ CFU/mL) were diluted 1:100 in fresh LB broth. Aliquots (200 μL) of each bacterial suspension were dispensed into 96-well flat-bottom microtiter plates. Growth kinetics were monitored at 37°C for 16 h in a microplate spectrophotometer, with OD_600_ measurements recorded at 20-min intervals. Five biological replicates were performed for each strain to ensure reproducibility

### String test

The string test was performed to assess hypermucoviscosity by culturing isolates overnight on LB agar at 37°C. A result was considered positive if stretching a colony with a sterile loop produced a mucoid thread > 5 mm long [23].

### Mucoviscosity assay

The bacterial cultures were grown overnight in LB broth and centrifuged for 5 min at 2000 rpm. Supernatant (200 μL) was placed in 96-well polystyrene plates and the OD_600_ was measured [24].

### Mouse infection model

Five-week-old female BALB/c mice were purchased from the Laboratory Animal Center of Hangzhou Medical College and maintained under specific pathogen-free (SPF) conditions. The animal assays were approved by the Institutional Animal Care and Ethics Committee at The First Affiliated Hospital of Zhejiang University, School of Medicine (2022–835). Ten five-week-old female BALB/c mice from each group were injected intraperitoneally with 10^7^ CFU of the strains. The physical condition of each mouse was monitored every 12 h. The murine survival rate over five days was measured. We selected classical NTUH-K2044 (from a Taiwanese liver abscess case) as a ST23 hvKP isolate, and KP47434 as a ST11-KL64 CR-hvKP isolate as previously reported [25,26].

## Statistical analysis

All experiments were performed independently, with at least three replicates. Continuous variables, represented by the means ± SDs, were tested using Student’s t-test. The total area under the curve was calculated for analysis on growth curve. The mortality of the mice was statistically assessed via a Kaplan-Meier analysis and a log-rank test, using the software GraphPad Prism (version 9). A *p*-value < 0.05 (two-tailed) was considered significant.

## Results

Between 2019 and 2023, a total of 1069 non-repetitive CRKP isolates involved in bloodstream infections were collected from 72 sentinel hospitals distributed across 22 provinces. Subsequent analysis of the whole genomes with MLST revealed four ST23 isolates (0.37%), which were classified as members of the globally disseminated hypervirulent clone [27]. Based on Kleborate analysis, these four genomes were classified as hypervirulent strains (virulence score = 5), belonging to the K1 and O1 capsular types associated with heightened pathogenicity. These isolates were detected in two hospitals located in Shaanxi and Zhejiang provinces in 2021 and 2023, respectively. We retrieved and reviewed the medical records from local sites. All four patients were male, aged between 58 and 76 years, with underlying comorbidities (Table 1). Of these cases, three were hospital-acquired, while one was community-acquired.

**Table 1.** Clinical characteristics and treatment strategies for the patients with ST23 CRKP bloodstream infection.*

	Patient 1	Patient 2	Patient 3	Patient 4
Isolate ID	KP169509	KP169543	KP245861	KP245905
Region	Shaanxi	Shaanxi	Zhejiang	Zhejiang
Department	Neurosurgery	Neurosurgery	EICU	Department of Infectious Diseases
Time	2021 Sept 19	2021 Sept 16	2023 Oct 18	2023 Nov 11
Gender	male	male	male	male
Age (years)	73	76	58	69
Admission Diagnosis	Secondary Epilepsy, Postoperative Cerebral Glioma	Cerebral Hemorrhage	Multiple Traumas	Bloodstream infections, Retroperitoneal Abscess
Comorbidity	Hypertension	Hypertension, Coronary Heart Disease	Postoperative Gastric Stromal Tumor	Hypertension
Invasive Procedures	Urinary Catheterization	Urinary Catheterization	Decompressive craniectomy, Endotracheal Intubation, Urinary Catheterization	Retroperitoneal Abscess Drainage, Central Venous Catheterization
Site of Pathogen Isolation	Blood	Blood	Blood	Blood
CRKP Infections at Alternate Sites	–	–	–	Retroperitoneal abscess
Acquisition origin	HA	HA	HA	CA
Laboratory results				
WBC (10^9^ /L)	8.32	5.14	5.6	28
Neutrophils (%)	94.8	73.9	93	88
Hb (g/L)	116	129	103	131
PLT (10^9^ /L)	53	168	366	219
ALB (g/L)	36.7	28.5	33.7	29.7
ALT (U/L)	30	66	65	36
AST (U/L)	28	83	47	25
TBIL (mmol/L)	17.7	15.5	12.2	29.3
Cr (mmol/L)	66.98	64.06	43	137
CRP (mg/L)	78	89.7	24.36	277.74
PCT (ng/mL)	2.13	0.319	0.14	9.75
Empirical antibiotic treatment	CSL→BIA	CXM→IPM	CRO→CSL	ETP
Definitive antibiotic treatment	MEM+POL→POL+TGC→ MEM+POL+TGC	LVX→CIP	CZA	CZA+LVX→AMK+LVX
ICU admission	No	No	Yes	No
Hospitalization (days)	51	34	25	19
Primary Disease Outcome	Malignant brain tumor recurrence	Partial recovery (mild hemiparesis)	Died (due to severe traumatic brain injury and polytrauma)	Partial recovery
Infection Outcome	Partial recovery†	Complete recovery	Partial recovery†	Partial recovery

*CRKP, carbapenem-resistant *Klebsiella pneumoniae*; EICU, Emergency Intensive Care Unit; WBC, white blood cell count; Hb, hemoglobin; PLT, platelet count; ALB, albumin; ALT, alanine aminotransferase; AST, aspartate aminotransferase; TBIL, total bilirubin; Cr, creatinine; CRP, C-reactive protein; PCT, procalcitonin; HA, hospital-acquired infection; CA, community-acquired infection; AMK, amikacin; BIA, biapenem; CIP, ciprofloxacin; CSL, cefoperazone/sulbactam; CRO, ceftriaxone; CXM, cefuroxime; CZA, ceftazidime/avibactam; ETP, ertapenem; IPM, Imipenem; LVX, levofloxacin; MEM, meropenem; POL, polymyxin B; TGC, tigecycline.

†Partial recovery is defined as pathogen clearance with symptomatic improvement, despite infection markers remaining above normal ranges.

Patient 1, a 73-year-old man with a history of cerebral glioma surgery (October 2020), was admitted on 30 August 2021 for recurrent glioma, secondary epilepsy, and hypertension, and was found to have CRKP (KP169509) on blood culture on September 19. He received sequential combination therapy with polymyxin B, meropenem, and tigecycline, achieving blood culture clearance by October 1 and resolution of recurrent fever by 3 October 2021. Patient 2, a 76-year-old man with cerebral hemorrhage (left parietal lobe and lateral ventricle), hypertension, and coronary heart disease, was admitted on 7 September 2021 for sudden right-sided limb weakness and urinary incontinence. He developed fever on September 16 with CRKP (KP169543) isolated from blood cultures, prompting combination treatment with imipenem, ciprofloxacin, and levofloxacin. The fever resolved by September 23 and blood cultures cleared by September 25, with the patient discharged on October 11 showing symptom improvement (reduced headache, afebrile, no sputum) (Figure S1). Notably, the two patients cohabited the same neurosurgery ward, and their CRKP isolates (KP169509 and KP169543), collected within a 3-day interval, showed only two cSNP differences (Figure S2), strongly suggesting nosocomial transmission.

Patient 3, a 58-year-old man, was transferred to Lishui Municipal Central Hospital on 7 October 2023 following multiple traumas from a fall, presenting in coma with severe injuries including various intracranial hemorrhages (subdural, intracerebral, epidural hematomas), skull and multiple bone fractures (ribs, lumbar vertebrae, scapula). Admitted to Emergency Intensive Care Unit (EICU), he developed CRKP (KP245861) bacteremia on October 18, successfully treated with ceftazidime-avibactam, achieving blood culture clearance by October 27. Despite microbiological resolution, treatment was withdrawn on November 1 due to irreversible traumatic brain injury and polytrauma, with death confirmed at 28-day follow-up. Patient 4, a 69-year-old man, presented on 11 November 2023 with high fever (40°C), chills and severe abdominal pain, and was found to have CRKP (KP245905) bacteremia. CT imaging revealed a retroperitoneal abscess and bilateral pulmonary infiltrates with pleural effusions, demonstrating systemic dissemination. The patient underwent surgical drainage of the abscess on November 15, with pus cultures also positive for CRKP. Antibiotic therapy was escalated from ertapenem to combination therapy with ceftazidime-avibactam, levofloxacin, and amikacin. Blood cultures cleared by November 17, with symptom resolution and inflammatory marker normalization leading to discharge on November 30 on oral levofloxacin. The two strains (KP245861 and KP245905) showed no clonal relationship (124 cSNPs difference), demonstrating separate evolutionary trajectories and likely acquisition from different sources.

All ST23 isolates demonstrated multidrug resistance to various antibiotics, including cephalosporins, carbapenems, and aztreonam (Table 2). Notably, strains KP245861 and KP245905 demonstrated limited resistance to ertapenem (MIC range: 4 - 8 μg/mL), while maintaining susceptibility to imipenem (MIC: 0.5 μg/mL), meropenem (MIC: 1 - 2 μg/mL), and ceftazidime-avibactam (MIC: 0.25/4 μg/mL). To elucidate the carbapenem resistance mechanisms in ST23 CRKP strains, we characterized their antibiotic resistance gene profiles. Each of the four isolates carried between 5 and 14 antimicrobial resistance genes. KP169509 and KP169543 possessed extended-spectrum β-lactamase genes (*bla*_CTX-M-15_ and/or *bla*_SHV-12_) as well as the carbapenemase gene *bla*_NDM-1_, whereas KP245861 and KP245905 harbored *bla*_KPC-2_ (Table 3). While *Klebsiella pneumoniae* strains harboring *bla*_KPC-2_ typically exhibit resistance to both imipenem and meropenem, isolates KP245861 and KP245905 demonstrated an atypical susceptibility profile despite carrying this carbapenemase gene. To elucidate the molecular basis of this phenotypic discrepancy, we quantified *bla*_KPC-2_ expression using RT-qPCR. Our analysis revealed significantly downregulated transcriptional levels of *bla*_KPC-2_ in these strains compared to the conventional *bla*_KPC-2_-positive control ATCC BAA-1705 (*p* < 0.05) (Figure S3).

**Table 2.** Susceptibility of four ST23 CRKP strains to commonly used antibiotics (μg/mL) .*

Isolates	TZP	CSL	CRO	CAZ	CXM	FEP	MOX	ATM	IPM	MEM	ETP	CZA	CIP	LVX	AMK	GEN	SXT	FOS	POLB	TGC
KP169509	128/4	128/64	64	64	128	64	128	64	16	4	32	32/4	0.06	0.06	1	64	8/152	4	0.5	0.125
KP169543	128/4	128/64	64	64	128	64	128	64	8	4	32	32/4	0.06	0.06	2	64	8/152	4	0.5	0.25
KP245861	>128/4	128/64	32	16	>128	4	4	32	0.5	1	4	0.25/4	1	0.5	2	1	0.25/4.75	1	0.5	0.125
KP245905	>128/4	64/32	16	16	>128	4	4	32	0.5	2	8	0.25/4	0.008	0.125	1	0.5	0.25/4.75	64	1	0.25

*Resistance to antibiotics is indicated by brown, while intermediate susceptibility is shown in light brown. TZP, piperacillin/tazobactam; CSL, cefoperazone/sulbactam; CRO, ceftriaxone; CAZ, ceftazidime; CXM, cefuroxime; FEP, cefepime; MOX, moxalactam; ATM, aztreonam; IPM, imipenem; MEM, meropenem; ETP, ertapenem; CZA, ceftazidime/avibactam; CIP, ciprofloxacin; LVX, levofloxacin; AMK, amikacin; GEN, gentamicin; SXT, sulfamethoxazole/trimethoprim; FOS, fosfomycin; POLB, polymyxin B; TGC, tigecycline.

**Table 3.** Genomic characteristics of four ST23 CRKP strains.*

Isolates	VS	No. VGs	VP replicons	No. ARGs	CR plasmid replicons	CPMs	Other ARGs in CR plasmid	Chromosomal ARGs
KP169509	5	133	IncFIB_K37_/IncHI1B(pNDM-MAR)	14	IncFII_K2_ and IncX3	NDM-1 (IncFII_K2_), NDM-1 (IncX3)	IncFII_K2_: *ble, sul1, aadA2, dfrA12, aac(3)-IId, bla*_TEM-1_,*bla*_CTX-M-15_ IncX3: *ble, bla*_SHV-12_	*oqxB19, oqxA, bla*_SHV-11_,*fosA*
KP169543	5	133	IncFIB_K37_/IncHI1B(pNDM-MAR)	12	IncFII_K2_	NDM-1	*ble, sul1, aadA2, dfrA12, aac(3)-IId, bla*_TEM-1_,*bla*_CTX-M-15_	*oqxB19, oqxA, bla*_SHV-11_,*fosA*
KP245861	5	133	IncFIB_K37_/IncHI1B(pNDM-MAR)	6	IncFII_K34_	KPC-2	*qnrS1*	*oqxB19, oqxA, bla*_SHV-11_,*fosA*
KP245905	5	133	IncFIB_K37_/IncHI1B(pNDM-MAR)	5	IncFII_K34_	KPC-2	,	*oqxB19, oqxA, bla*_SHV-11_,*fosA*

*VS, virulence score, is calculated by the presence of the genes that encode aerobactin (three points), yersiniabactin (one point), and colibactin (one point); VGs, virulence genes; VP, virulence plasmid; ARGs, antibiotic resistance genes; CR, carbapenem-resistant; CPMs, carbapenemases.

The assembled sequence data of ST23 CRKP isolates have been deposited in the GenBank database under BioProject accession number PRJNA1206662.

**Figure 1.**
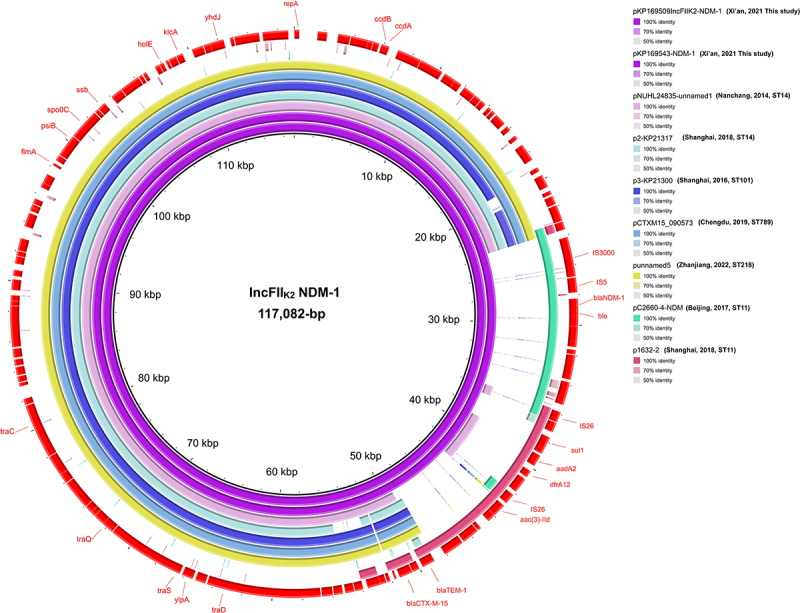
Circular comparison of the sequences between pKP169509IncFII_K2_-NDM-1, pKP169543-NDM-1, pNUHL24835-unnamed1 (CP014005.1), p2-KP21317 (CP124700.1), p3-KP21300 (CP124696.1), pCTXM15_090573 (CP071168.1), punnamed5 (CP101795.1), pC2660-4-NDM (CP039811.1), and p1632-2 (CP084499.1). The plasmid pKP169509IncFII_K2_-NDM-1 was used as the reference.

To resolve the genetic architecture of carbapenemase genes and characterize resistance plasmids, we conducted long-read sequencing of these ST23 isolates. The clonally related strains KP169509 and KP169543 both carried an IncFII_K2_ NDM-1 plasmid, namely the 117,082-bp pKP169509IncFII_K2_-NDM-1 and the 117,082-bp pKP169543-NDM-1, respectively. Similarly, the genetic structure of the *bla*_NDM-1_ gene on these plasmids was organized as *IS3000-IS5-bla*_NDM-1_-*ble-trpF*. Both plasmids were successfully transferred to *Escherichia coli* EC600 and carried a multidrug resistance (MDR) region encompassing *sul1*, *aadA2*, *dfrA12*, *aac(3)-IId*, *bla*_TEM-1_, and *bla*_CTX-M-15_ (Figure 1). Comparative plasmid analysis identified genomic conservation (>99% sequence identity, 80–83% coverage) between these IncFII_K2_ plasmids and five historically IncFII_K2_ variants in China: pNUHL24835-unnamed1 (2014; CP014005.1), p2-KP21317 (2018; CP124700.1), p3-KP21300 (2016; CP124696.1), pCTXM15_090573 (2019; CP071168.1), and punnamed5 (2022; CP101795.1) (Figure 1). This striking genomic conservation, maintained consistently over an eight-year epidemiological timeframe (2014–2022), strongly suggests both (i) evolutionary descent from a pNUHL24835-unnamed1-like ancestral plasmid and (ii) sustained dissemination of this stable plasmid backbone among clinical *K. pneumoniae* populations in China. Notably, two distinct acquired regions were identified: an NDM-1 resistance cassette showing > 99% identity to pC2660-4-NDM (2017; CP039811.1) and a multidrug resistance region (*sul1-aadA2-dfrA12-aac(3)-IId*) with >99% identity to p1632-2 (2018; CP084499.1) (Figure 1). Comparative genomic analysis revealed KP169509 harbored a conjugative IncX3 plasmid (pKP169509-IncX3-NDM-1) containing *bla*_NDM-1_ and *bla*_SHV-12_, absent in KP169543, highlighting plasmid dynamics in the evolution of this nosocomial strain. The *bla*_NDM-1_ gene was embedded in a similar genetic context of *IS3000-IS5-bla*_NDM-1_-*ble-trpF*. It shared 99.99% identity with Chinese enterobacterial IncX3 plasmids pNDM1_020135 (CP037965.1), pHZKP1 (CP139934.1), pBSI034-NDM1 (MN937240.1), and pEk72-3 (CP088232.1), with 100% query coverage (Figure S4).

In contrast, both KP245861 and KP245905 harbored a conjugative IncFII_K34_ KPC-2 plasmid, namely the 103,159-bp pKP245861-KPC-2 and the 104,960-bp pKP245905-KPC-2, respectively. The IncFII_K34_ KPC-2 plasmid has been previously implicated in the global dissemination of ST23 CR-hvKP [11,28]. In both plasmids, the *bla*_KPC-2_ gene was located within a conserved genetic structure (*ΔISKpn6-bla*_KPC-2_-*ISKpn27-tnpR-IS26*). pKP245861-KPC-2 exhibited 99.92% identity and 94% coverage with pKP245905-KPC-2, with the primary difference being a ~ 6 kb region containing the *qnrS1* gene. Sequence alignment analysis revealed that these plasmids shared 99.92%–100% identity with Chinese enterobacterial plasmids pKPC-DD02357 (CP096236.1), p2838-KPC (CP047686.1), and pHS1446 (OR805038.1), with query coverages ranging from 93% to 100% (Figure S5).

We further compared the conjugation rates and fitness costs of these carbapenemase-encoding plasmids. Due to the coexistence of two distinct conjugative NDM-1-encoding plasmids in KP169509, the individual conjugation frequency of each plasmid could not be precisely delineated for this isolate. Notably, while the IncFII_K34_ KPC-2 plasmid has been reported to exhibit high conjugation efficiency [11], our experiments revealed that the transfer efficiency of the IncFII_K2_ NDM-1 plasmid pKP169543-NDM-1 from clinical CR-hvKp isolates to *E. coli* EC600 recipient strains was moderately lower [(7.73 ± 6.40) ×10^−5^] compared to the IncFII_K34_ KPC-2 plasmids pKP245861-KPC-2 [(4.87 ± 3.91) ×10^−4^] and pKP245905-KPC-2 [(4.78 ± 2.72) ×10^−4^] on LB agar, with this difference not reaching statistical significance (*p* > 0.05) (Figure S6A). Interestingly, when tested on LB agar supplemented with meropenem (0.01 μg/mL), the conjugation efficiency of pKP169543-NDM-1 [(8.03 ± 1.27) ×10^−5^] was significantly lower than pKP245861-KPC-2 [(8.28 ± 2.37) ×10^−4^] and pKP245905-KPC-2 and [(9.45 ± 2.16) ×10^−4^] (*p* < 0.05) (Figure S6B). Fitness assessment revealed that the EC600:pKP169543-NDM-1 transconjugant exhibited similar growth characteristics to both KPC-2-bearing transconjugants (EC600:pKP245861-KPC-2 and EC600:pKP245905-KPC-2), and all plasmid-carrying variants showed comparable fitness compared to the wild-type EC600 strain (*p* > 0.05) (Figure S7). These findings collectively highlight the significant contribution of both IncFII_K2_ NDM-1 and IncFII_K34_ KPC-2 plasmids as crucial vehicles for the spread of antimicrobial resistance, with the IncFII_K34_ KPC-2 plasmid showing superior conjugation efficiency particularly under antibiotic pressure. To further investigate the prevalence of IncFII_K2_ NDM-1 plasmids, we analyzed all complete IncFII_K2_ plasmids (*n* = 590) available in PLSDB (version 2024_05_31_v2) [29]. Only 26 plasmids (4.4%) were found to carry the *bla*_NDM-1_ gene. Bioinformatic analysis predicted all 26 plasmids to be conjugative, with their host distribution exclusively limited to Enterobacteriaceae: *Klebsiella pneumoniae* (*n* = 21), *Klebsiella variicola* (*n* = 1), *Escherichia coli* (*n* = 2), *Enterobacter hormaechei* (*n* = 1), and one unidentified Enterobacteriaceae strain (Table S1). Geospatial analysis revealed distinct distribution patterns: the United States represented the most common origin (*n* = 12), followed by Thailand (*n* = 3). Only two plasmids were detected in mainland China and Taiwan (one each). Among the 21 *Klebsiella pneumoniae* strains harboring IncFII_K2_ NDM-1 plasmids, 17 had genome assemblies available in the NCBI database. Genomic analysis revealed considerable clonal diversity, with ST133 (*n* = 4), ST14 (*n* = 4), and ST219 (*n* = 3) representing the most prevalent sequence types (Table S1). Notably, none of the IncFII_K2_ NDM-1 plasmid-positive strains belonged to the ST23 clone.

**Figure 2.**
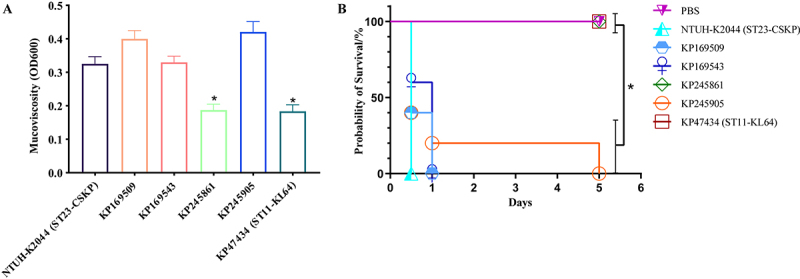
Virulence potential of ST23 isolates in the study. (A) Centrifugation analysis of the strains.

To characterize the pathogenic potential of ST23 CRKP strains, we analyzed both virulence factor profiles and phenotypic virulence characteristics in *Klebsiella pneumoniae*. All strains harbored an identical virulence gene profile and carried a conserved IncFIB_K37_/IncHI1B(pNDM-MAR) virulence plasmid encoding *rmpA*, *rmpA2*, *iucA*, and *iroB* (Table S2). Notably, KP169509 and KP169543 contained a 30-kb deletion in their virulence plasmids. This deleted region lacked known virulence determinants but included multiple hypothetical protein-coding sequences (Figure S8). The hypermucoviscous phenotype was confirmed in three of the four ST23 CRKP strains via a positive string test (viscous string > 5 mm), whereas KP245861 tested negative. We further performed the sedimentation test to evaluate the hypermucoviscous phenotype. The results demonstrated that KP245861 exhibited significantly lower mucoviscosity compared to the other ST23 CRKP strains (*p* < 0.05) (Figure 2A). Given the crucial role of capsule in determining mucoviscosity [30], we hypothesized that the observed low mucoviscosity and non-hypermucoviscous phenotype resulted from mutations in either the capsular polysaccharide biosynthesis (*cps*) locus or *rmpA/A2* regulatory genes. Comparative genomic analysis of KP245861 against reference strain NTUH-K2044 and KP245904 identified two key mutations: S580T (TCA→ACA) in the tyrosine autokinase Wzc and K329E (AAA→GAA) in the glycosyltransferase WcaJ. These non-synonymous mutations likely impair capsule production, thereby attenuating virulence. Supporting this hypothesis, *in vivo* infection experiments demonstrated significantly lower mortality rates for KP245861 and CR-hvKP strain KP47434 compared to NTUH-K2044 and three ST23 CRKP strains (*p* < 0.05) (Figure 2B). These findings indicate that while KP245861 lost hypervirulence, other ST23 CRKP strains maintained their hypervirulent phenotype despite carbapenem resistance.

(B) Murine survival curves in the intraperitoneal infection model. We selected classical NTUH-K2044 (from a Taiwanese liver abscess case) as a ST23 hvKP isolate, and KP47434 as a ST11-KL64 CR-hvKP isolate. *, *p* < 0.05.

## Discussion

With growing clinical concern regarding the convergence of carbapenem resistance and hypervirulence in *Klebsiella pneumoniae*, two distinct evolutionary pathways have emerged: (1) CRKP acquiring virulence plasmids (e.g. the epidemic ST11-KL64 clone in China), generating hypervirulent CRKP strains with moderately enhanced virulence that remains below classical hvKP levels [31]; and (2) classical hvKP lineages (e.g. ST23-K1) incorporating carbapenemase plasmids, forming CR-hvKP. While the thick capsule of hvKP naturally restricts plasmid uptake, successful resistance acquisition in these strains frequently coincides with capsular polysaccharide gene mutations that attenuate virulence [6,7]. Although ST23 CR-hvKP has become increasingly prevalent in the current *Klebsiella pneumoniae* genomic landscape, comprehensive genomic surveillance remains crucial to identify novel plasmid types and carbapenemase variants in these strains. Such efforts are essential for elucidating the complex interplay between resistance and virulence evolution.

Our national surveillance system BRICS identified four ST23 CRKP strains causing bloodstream infections. Genomic analysis revealed a nosocomial transmission cluster involving two ST23 CRKP isolates originating from the same neurosurgery department in a Shaanxi hospital. Carbapenemase gene distribution showed regional specificity, with *bla*_NDM-1_ and *bla*_KPC-2_ each detected in two strains – contrasting the predominant *bla*_OXA-48_-bearing ST23 CR-hvKP strains reported in Europe and Taiwan [8,32]. Notably, two *bla*_KPC-2_-positive isolates (KP245861 and KP245905) exhibited an atypical susceptibility profile, maintaining susceptibility to both imipenem and meropenem. This phenomenon aligns with previous reports demonstrating delayed development of high-level carbapenem resistance in K1/K2 hvKP strains following KPC plasmid acquisition [6,33,34]. Given the established correlation between *bla*_KPC-2_ expression levels and resistance phenotypes [35,36], we performed RT-qPCR analysis which revealed significantly downregulated *bla*_KPC-2_ transcription in these strains compared to the *bla*_KPC-2_-positive control ATCC BAA-1705. These findings suggest potential suppression of carbapenemase expression in hypervirulent backgrounds, though the precise regulatory mechanisms require further investigation.

Through comprehensive genomic characterization, we elucidated the genetic architecture of carbapenemase genes and their associated resistance plasmids in ST23 CRKP strains. Our findings align with recent work by Jiang et al. demonstrating the crucial evolutionary role of IncFII_K34_ KPC-2 plasmids in ST23 CR-hvKP [11], as evidenced by strains KP245861 and KP245905 harboring this plasmid type. Notably, while an IncFII_K2_ KPC-2 plasmid was previously reported in a U.S. ST23 CR-hvKP isolate [9], we provide the first documentation of a conjugative IncFII_K2_ NDM-1 plasmid in a hospital-transmitted ST23 CR-hvKP clone – a concerning discovery given this plasmid’s high conjugation capacity yet previously unrecognized association with ST23 CR-hvKP emergence. Whole-plasmid comparative genomic analysis indicates these IncFII_K2_ NDM-1plasmids likely evolved from a pNUHL24835-unnamed1-like ancestor through sequential acquisition of: (i) an NDM-1-bearing genetic fragment (showing > 99% identity to pC2660-4-NDM), and (ii) a multidrug resistance cluster (*sul1-aadA2-dfrA12-aac(3)-IId*) homologous to p1632-2. Bioinformatic analysis revealed all characterized IncFII_K2_ NDM-1 plasmids maintain conjugative potential and demonstrate exclusive Enterobacteriaceae tropism, underscoring their significant role in resistance dissemination. These findings demonstrate the important role of plasmids in driving resistance-virulence convergence in CR-hvKP and highlight the need for enhanced plasmid surveillance alongside traditional clonal tracking to better anticipate and prevent the emergence of such high-risk strains.

The evolutionary trajectory of CR-hvKP underscores the intricate balance between antimicrobial resistance and virulence in the post-antibiotic era [28]. Unlike CRKP strains acquiring virulence plasmids, hvKP strains incorporating KPC plasmids rarely maintain both hypervirulence and carbapenem resistance simultaneously [6]. To assess this phenomenon, we systematically evaluated the pathogenic potential of our isolates. All strains harbored a conserved IncFIB_K37_/IncHI1B(pNDM-MAR) virulence plasmid with identical virulence gene profiles. However, strain KP245861 exhibited reduced mucoviscosity and failed to demonstrate the characteristic hypermucoviscous phenotype. Comparative genomic analysis revealed two non-synonymous mutations in the *cps* locus: S580T (TCA→ACA) in *wzc* (encoding polysaccharide biosynthesis tyrosine autokinase) and K329E (AAA→GAA) in *wcaJ* (encoding undecaprenyl-phosphate glucose-1-phosphate transferase). Mutations in these genes, previously associated with altered capsule production and virulence attenuation [30,37,38], likely account for the observed phenotypic differences. *In vivo* infection models confirmed KP245861’s attenuated virulence, while other ST23 CRKP strains maintained hypervirulence despite carbapenem resistance. Notably, three ST23 CR-hvKP strains exhibited significantly higher mortality rates compared to the ST11 CR-hvKP strain KP47434. Currently, ST11 has emerged as the predominant clone of CR-hvKP in China, responsible for approximately 60–80% of clinical cases, with its carbapenem resistance primarily mediated by KPC-type carbapenemases [2,39]. However, our *in vivo* infection model data demonstrate that most ST23 CR-hvKP strains exhibit significantly greater virulence potential than ST11 CR-hvKP, showing higher mortality rates in mice within the same observation period. This suggests that ST23 infections may progress more rapidly in clinical settings, particularly in cases of delayed treatment or limited drug availability, potentially leading to worse outcomes.

This investigation has several limitations that should be acknowledged. Firstly, our phenotypic characterization was limited to four clinical ST23 isolates harboring either IncFII_K34_ KPC-2 or IncFII_K2_ NDM-1 plasmids. These isolates were identified through the national BRICS surveillance program, which specifically monitors antimicrobial resistance patterns in bloodstream infections. Consequently, our analysis did not include systematic sampling of isolates from other anatomical sites or infection types. This focused sampling approach may potentially underestimate the true prevalence and clinical distribution of these plasmid types across different infection contexts; Secondly, the precise regulatory mechanisms underlying the suppression of carbapenemase expression in two *bla*_KPC-2_-positive isolates (KP245861 and KP245905) remain unclear. While we observed reduced *bla*_KPC-2_ expression in these clinical isolates, the molecular basis for this phenomenon requires further investigation; Thirdly, we acknowledge that this was a retrospective study, and no environmental sampling (e.g. hospital surfaces or medical equipment) or screening of potential human reservoirs (other patients or healthcare workers) was conducted during the investigation period. Consequently, we lack additional microbiological evidence to definitively confirm nosocomial transmission.

In conclusion, this study provides the first genomic evidence of a conjugative IncFII_K2_ NDM-1 plasmid in a hospital-transmitted ST23 CR-hvKP clone. Implementation of plasmid-focused genomic surveillance, coupled with rapid diagnostic tools targeting resistance-virulence hybrids, will be crucial for controlling their spread in clinical settings.

## Supplementary Material

Figure S7.jpeg

Figure S8.jpeg

Author Checklist .pdf

Figure S1.tif

Figure S6.jpeg

Figure S5.jpeg

Figure S2.tif

Figure S4.jpeg

Figure S3.jpeg

## Data Availability

The complete genome sequences of four ST23 clinical isolates have been deposited in the NCBI database under BioProject PRJNA1206662, with individual BioSample accession numbers SAMN46080746-SAMN46080749. All supplementary materials, including Tables S1 and S2, along with additional datasets generated during this study, are publicly available through the Figshare repository (DOI: https://doi.org/10.6084/m9.figshare.28334051) [40]. This study was conducted in accordance with the ARRIVE guidelines (Animal Research: Reporting of *In Vivo* Experiments). The completed ARRIVE checklist has been included as part of the supplementary materials accompanying this article.
