# Supplementary figures and images for: Genomic analyses identify nosocomial transmission of ST23 carbapenem-resistant hypervirulent Klebsiella pneumoniae mediated by a conjugative IncFIIK2 NDM-1 plasmid
Source: Virulence. 2026 May 3;17(1):2668167. doi: 10.1080/21505594.2026.2668167 (PMC13138083; doi:10.1080/21505594.2026.2668167)

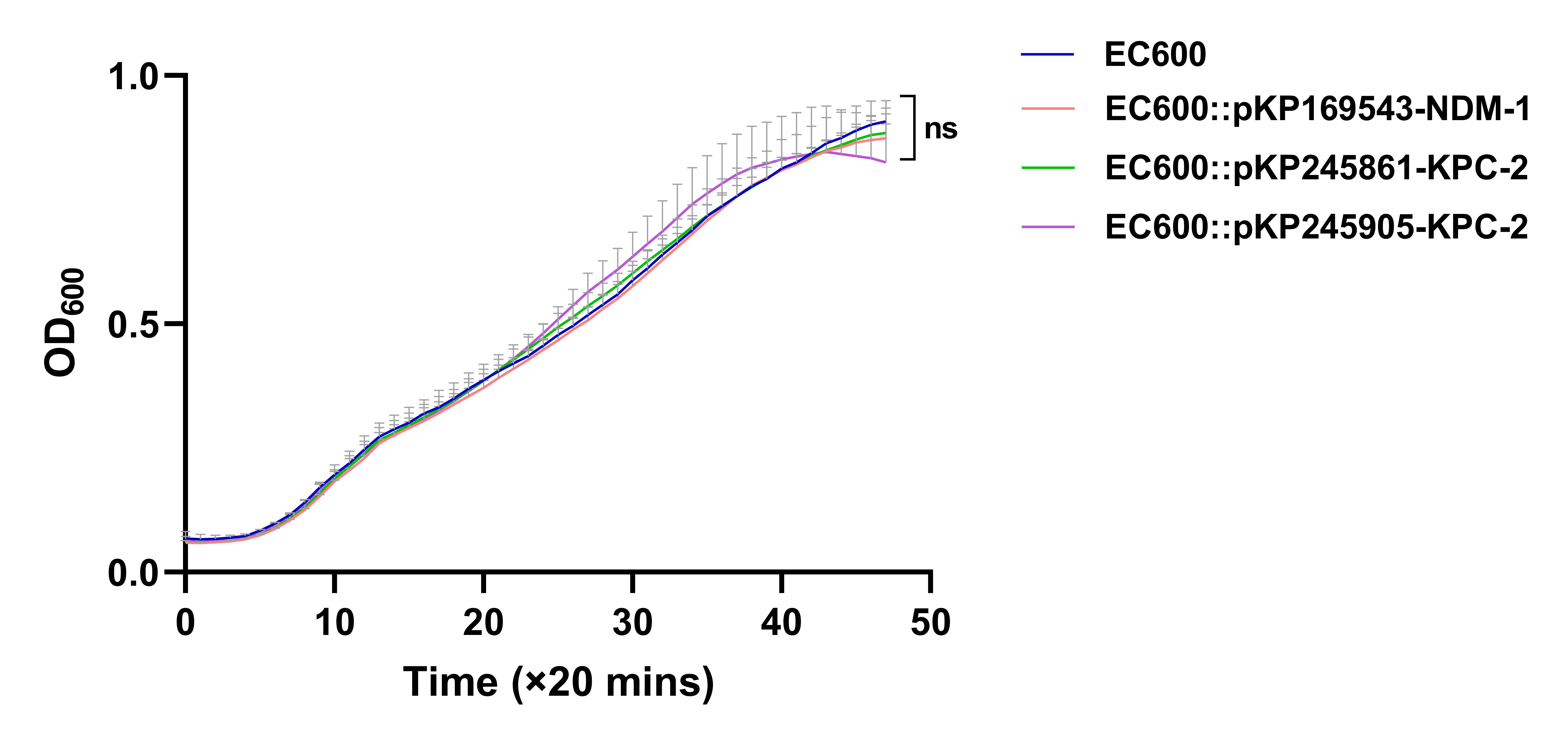

Supplement: Figure S7.jpeg [file KVIR_A_2668167_SM2873.jpeg]

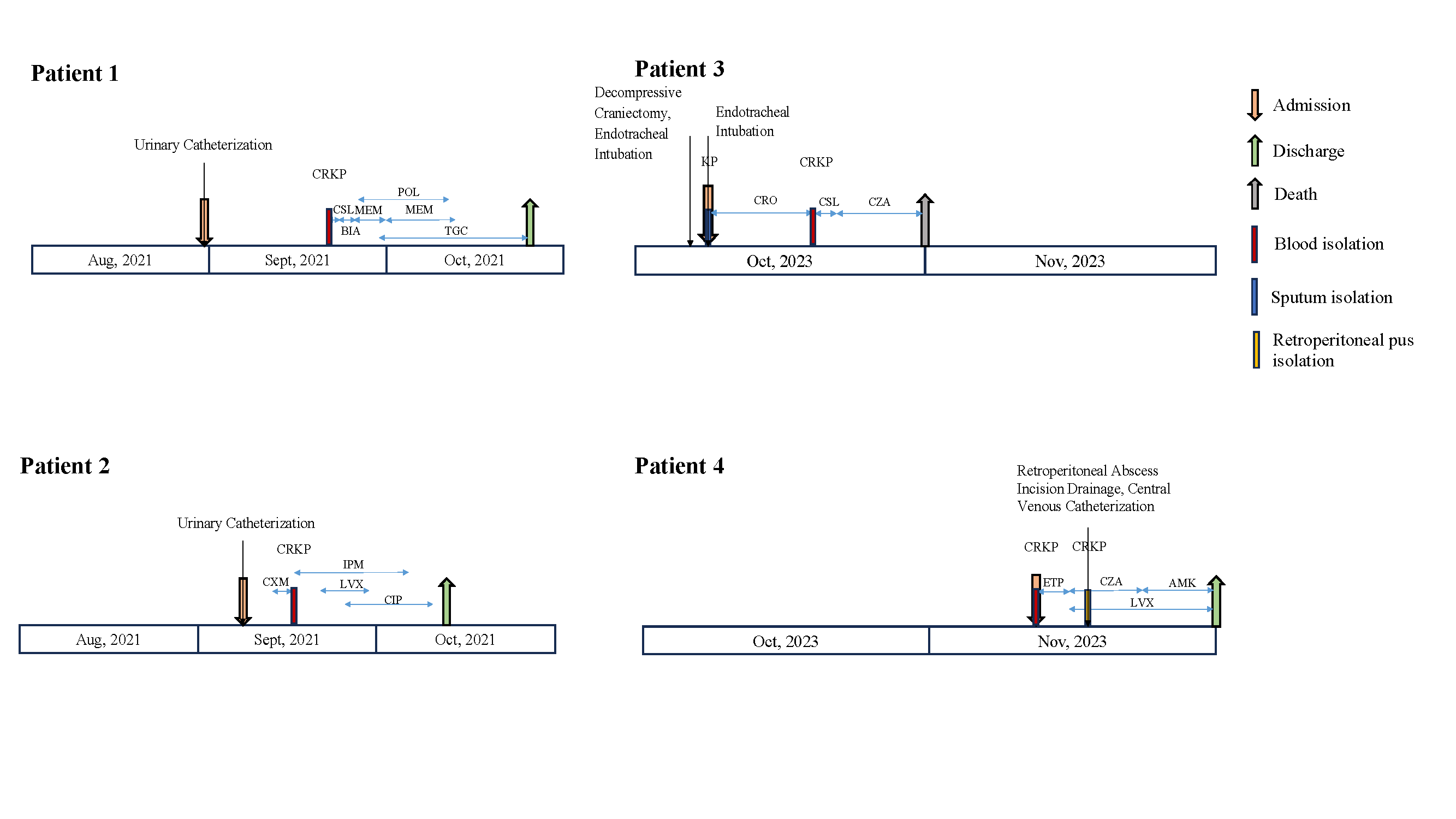

Supplement: Figure S1.tif [file KVIR_A_2668167_SM2870.tif]

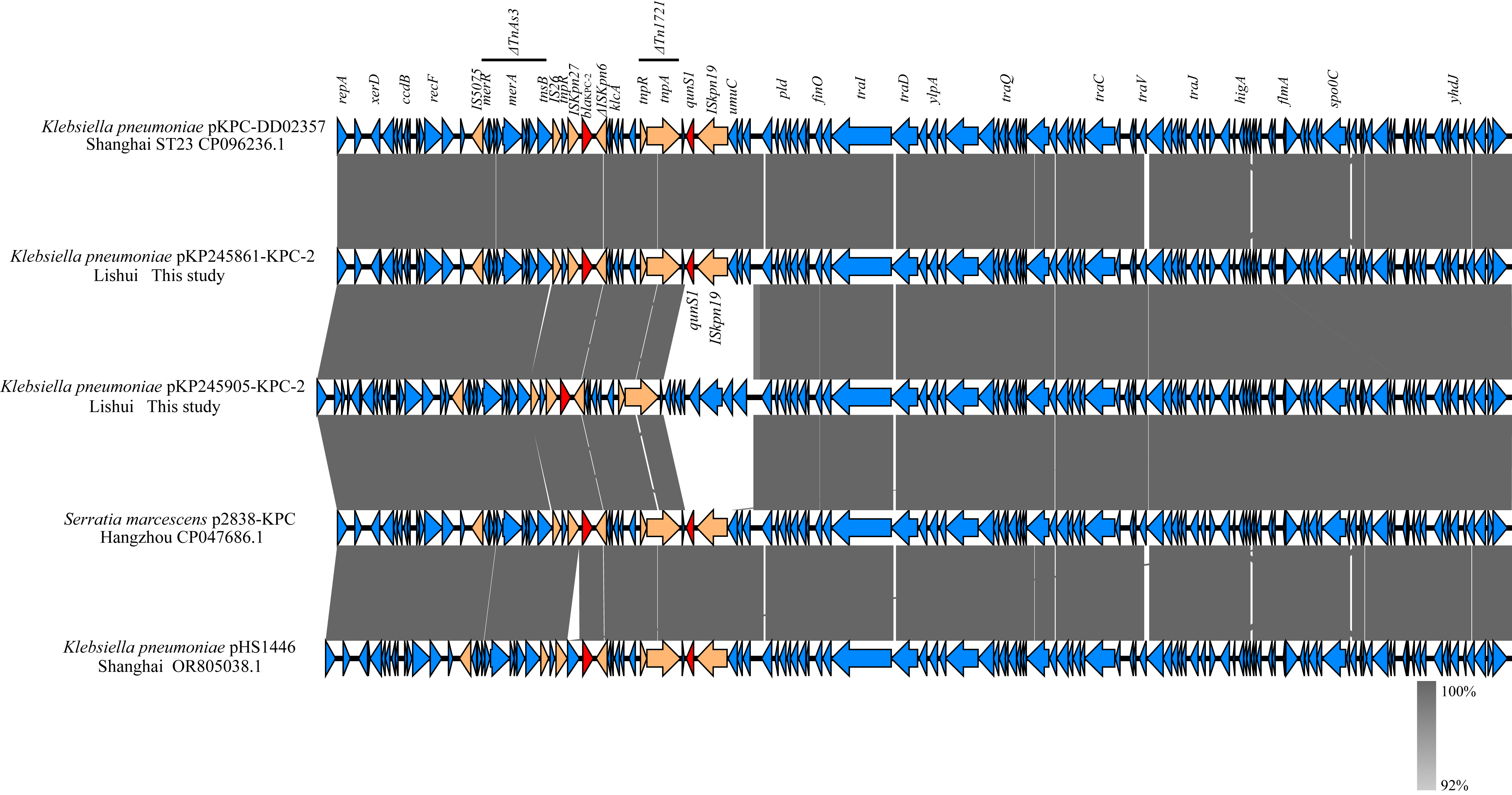

Supplement: Figure S5.jpeg [file KVIR_A_2668167_SM2868.jpeg]

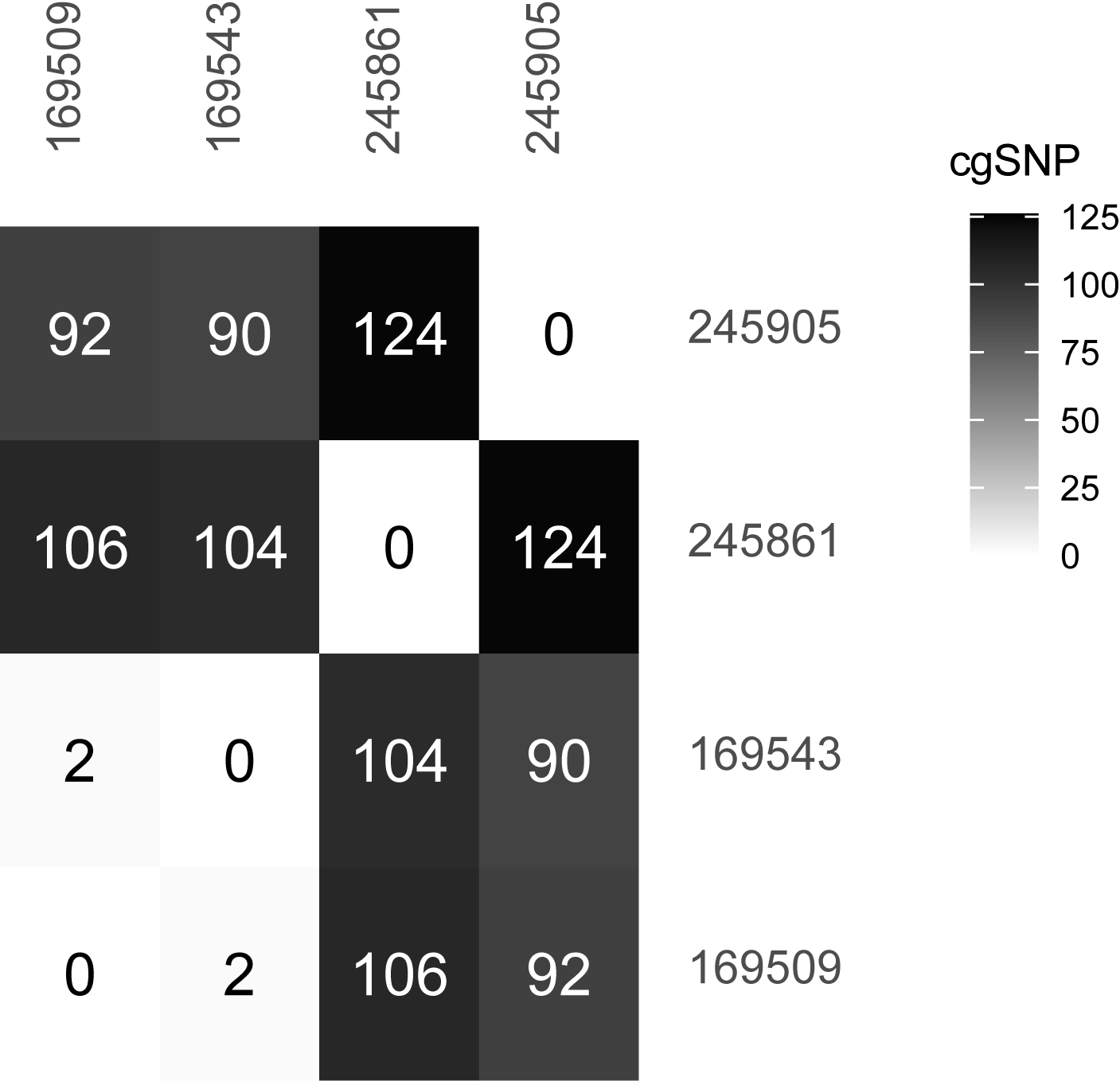

Supplement: Figure S2.tif [file KVIR_A_2668167_SM2867.tif]

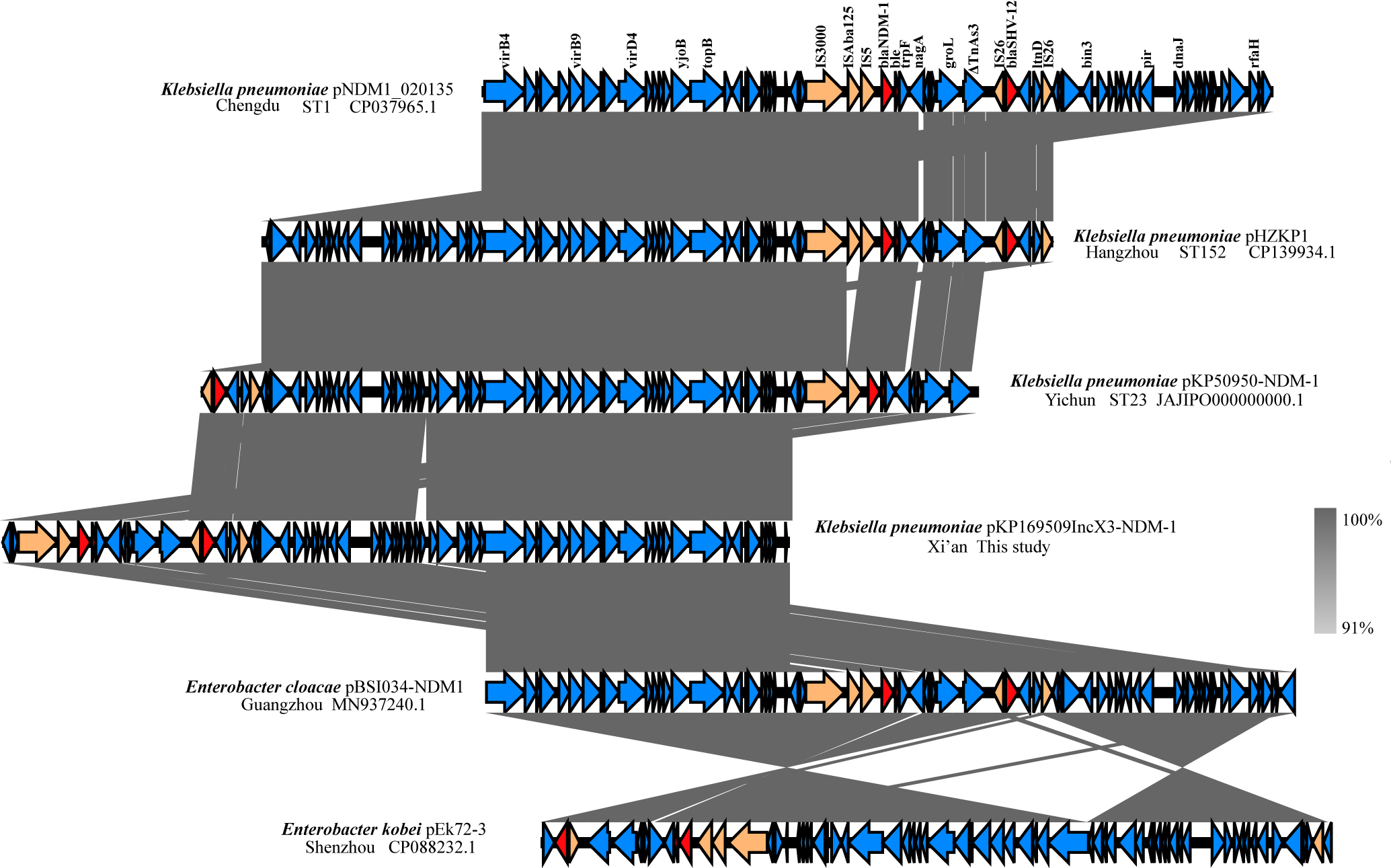

Supplement: Figure S4.jpeg [file KVIR_A_2668167_SM2866.jpeg]

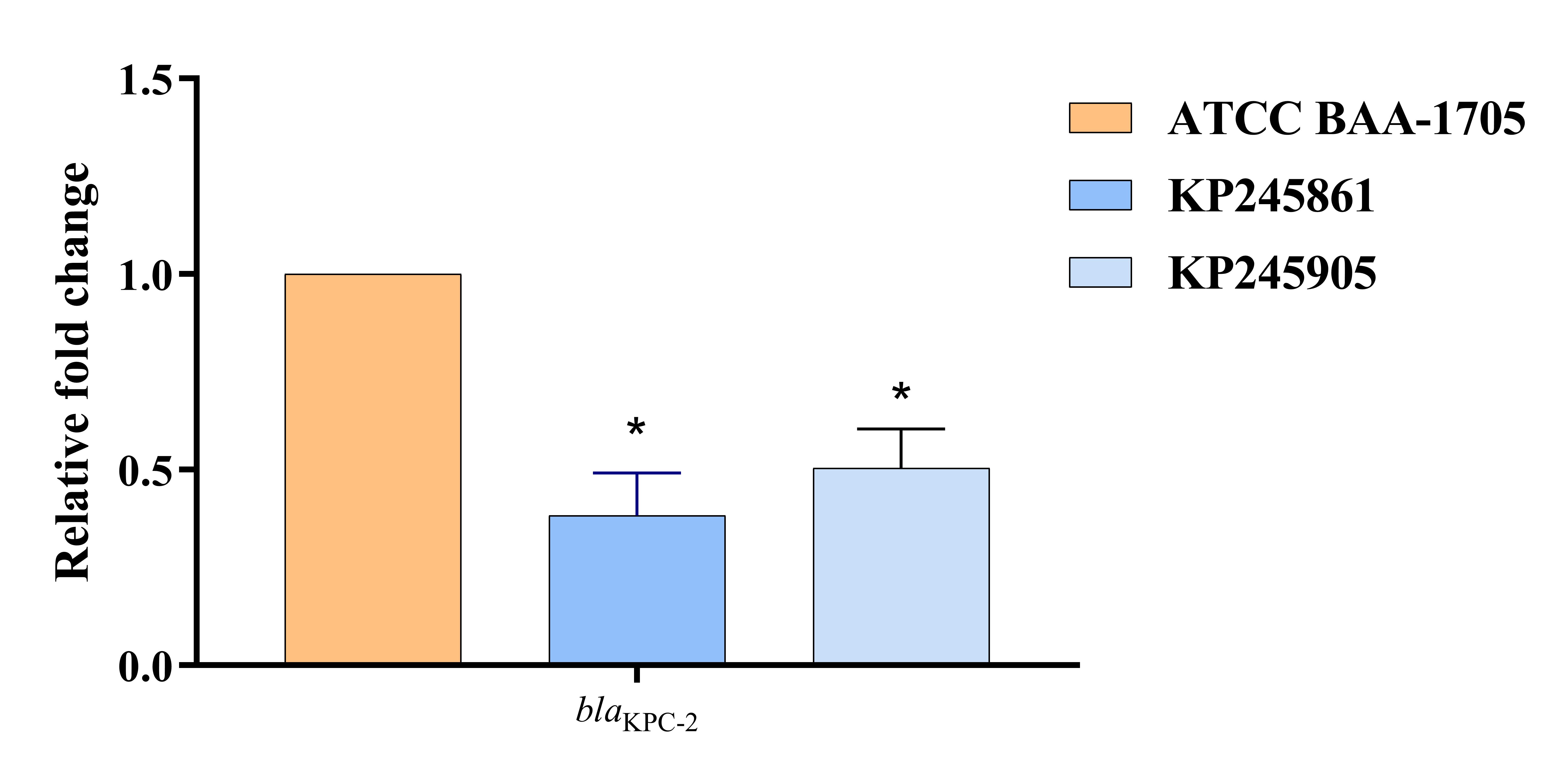

Supplement: Figure S3.jpeg [file KVIR_A_2668167_SM2865.jpeg]
